# Knowledge, attitude and practice among non-ophthalmic health care providers regarding eye management of diabetics in private sector of Riyadh, Saudi Arabia

**DOI:** 10.1186/s12913-019-4216-9

**Published:** 2019-06-13

**Authors:** Talal Bassam Abu-Amara, Waleed Abdulaziz Al Rashed, Rajiv Khandekar, Hamad Mohammed Qabha, Faris Mohammad Alosaimi, Abdulrahman Abdullah Alshuwayrikh, Mansour Khalid Almadi, Alanoud Alfaris

**Affiliations:** Department of Ophthalmology, Imam Mohammad Bin Saud Islamic University, collage of medicine, Riyadh, Saudi Arabia

**Keywords:** KAP, Diabetes, Diabetic retinopathy, Primary health care providers, Screening

## Abstract

**Background:**

The levels of knowledge, attitude and practice among primary physicians concerning both diabetic retinopathy screening and treatment of sight threatening diabetic retinopathy have been studied by different groups, such as medical students, pharmacists, Primary Health Care staff and opticians. In some studies, the levels were very high, while in others it was noted to be less than desired.

**Aim:**

This study’s intent is to estimate and improve level of Knowledge (K), Attitude (A) and Practice (P) among non-ophthalmic health care providers regarding eye management of diabetes and barriers that people with diabetes face in Saudi Arabia.

**Methods:**

This cross-sectional survey targeted medical doctors (except ophthalmologists) working at private sector institutions in Riyadh. They were interviewed using closed-ended questions for knowledge (8), attitude (5), practice (5), and reasons for their current KAP status comprised of 8 questions. The level of Knowledge was assessed as good if its score was (> 50%); positive attitude (> 50%) and excellent practice (> 75%) were estimated and associated to the risk factors.

**Results:**

Out of the 355 participants that were interviewed, the percentages of good knowledge, positive attitude and excellent practice concerning diabetic retinopathy (DR)were 193 [54.3% (95% CI 49.2–59.5)], 111 [31.3% (95% CI 26.4–36.1)], and 145 [40.8% (95% CI 35.7–46.0) participants, respectively. Gender, place of work and type of doctor were not significantly associated with the level of KAP. Salient reasons for low KAP status included a busy schedule (54.6%), less resources (75.2%), inadequate periodic training in eye care (69%), and absence of retinal evaluation training (49.6%).

**Conclusions:**

Improving KAP level is urgently needed. Addressing underlying causes of low KAP could enhance eye care of people with diabetes. Additionally, training for primary health care providers for early detection of DR and timely management of sight threatening diabetic retinopathy (STDR) is necessary.

## Background

Physicians and family physicians are the first level health contacts for people with diabetes. Therefore, they are crucial for primary prevention-related counselling as well as proper guidance for further care by experts who deal with the systemic complications of diabetes [1]. In countries like Saudi Arabia, where both diabetes mellitus (DM) and diabetic retinopathy (DR) is of epidemic proportion [2, 3], program approaches to deal with diabetes and diabetic retinopathy become of paramount importance [4]. Proper knowledge and the positive mind set of these health care providers could have a cascading effect in improving the practices both of caregivers and the adherence of people with diabetes to the diabetic care advice given to them [5]. Increasing private sector involvement in health care across the kingdom is evident. There were 1104 and 891 non-ophthalmic health care providers in private and governmental sector institutions of Riyadh respectively in 2016 [6].

Consequently, research about perspective of care providers should also focus on those working in the private sector due to their greater prevalence. The levels of knowledge, attitude and practice among Primary healthcare (PHC) physicians concerning both DR screening and treatment of sight-threatening diabetic retinopathy have been studied by different groups such as medical students [7], pharmacists [8], PHC staff [9] and opticians [10]. In some studies, the levels were very high while, in others, they were noted to be less than desired.

In addition to governmental institutions, the diabetic care in the study area is provided by both Saudi and non-Saudi health care providers in private institutions. Therefore, information of KAP of private sector primary physicians would complement about current status of non-ophthalmic care providers of people with diabetes to a large extent.

To the best of our knowledge KAP and barriers for eye care related to the knowledge and practice among private sector primary physicians have not been studied in central Saudi Arabia. Consequently, this study was conducted to assess the level of knowledge (K), attitude (A), and practice (P) among non-ophthalmic health care providers. Additionally, its intent was to improve the awareness of the early detection of DR and the timely management of sight-threatening diabetic retinopathy (STDR) in this group.

## Methods

This cross-section type of study was undertaken between June and September 2017 and was approved by the Institution Review Board (IRB) of the Al Imam Mohammad Bin Saud Islamic University, Riyadh, Saudi Arabia [NO. 0014/12/016/31]. Doctors of randomly selected health institutions from the private sector, unrelated to secondary level eye care services, were invited to participate in this survey. Their verbal consent to participate in the survey was obtained.

We assumed that acceptable level of practice for DR screening was 53% in primary care physicians [9]. In the study area, there were 1100 non-ophthalmic primary health care professionals. The target was to achieve 95% confidence interval with a 5% acceptable error margin and 1.2 factor for clustering. The final sample size was comprised of 341 randomly selected professionals.

A health educator, five medical students and three ophthalmologists conducted this study. A pretested questionnaire was used to collect information regarding participant demographics, physician medical education and the doctors’ interaction with patients during routine work in the work station. There were eight questions related to knowledge of DR diagnosis and management. The participants were given different options for response. If the reply of participants matched to the correct answer, per a panel of three ophthalmologist experts, 2 points were awarded. If the answer was incorrect, the participant received - 2 point. If a participant did not attempt to answer, 0 point were given. The sum of all responses for knowledge related question were further graded as: excellent, if the score was > 75% of the total possible score; good, if the score was between 51 and 75% and Poor, if the score was between 26 to 50%. A participant having a score 25% or less was considered to have very poor knowledge of DR.

Next, there were five questions related to the attitude towards DR screening and its management. The responses were based on five graded Likert scale. The total score of the responses for attitude-related question were grouped as positive attitude if score was > 50% of the total correct responses, per the expert. If it was < 50%, the attitude of the participant was considered negative.

Finally, there were five questions to inquire about the current practices for DR screening and DR management among participants while dealing with their patients. If the response of the participant matched to that of the expert panel, 2 points were allotted. If the answer was wrong, − 2 points were awarded and for those who did not attempt the question, a 0 score was recorded. The total sum was further separated into excellent (> 75% score), good (50 to 75%), poor (25 to 49%) and very poor (< 25%).

The feedback of the participants was collected for improving the eye care of people with diabetes and the possible barriers that doctors face in improving their KAP regarding DR.

The data was collected using an English version of questionnaire. The responses were transferred into a spread sheet of Microsoft Excel®. A univariate analysis, using the parametric method was carried out with the help of Statistical Package for Social Sciences (SPSS 24, IBM, Chicago, USA). The frequency and percentage proportions of the participants with excellent and good levels of knowledge, positive attitudes and acceptable (excellent + good) level of practice was associated to the demographic, medical education level and work-related variables. A two-sided *P*-value of < 0.05 was considered statistically significant.

## Results

### Demographic characteristics

The present study recruited 355 non-ophthalmic health care providers. Four participants opted not to contribute to the survey after recruitment. The median age of the participants was 39 years-of-age (25% quartile = 32 years-of-age) with a median of 13 years’ experience after graduation (25% quartile = 7 years-of-experience). Two-thirds of the participants (*n* = 238, 67%) were male, and the majority were non-Saudi (*n* = 352, 99.2%). General practitioners constituted most of the enrolled physicians (*n* = 182, 51.3%), followed by internists (*n* = 115, 32.4%). More than two-thirds of the physicians were working in a polyclinic (*n* = 255, 71.8%). Furthermore, one in ten physicians (*n* = 36) stated that they encountered diabetes in more than 50% of their patients. More demographic characteristics are given in Table 1.

**Table 1:** Profile of participating non-ophthalmic health care providers in private sector of Riyadh

Age	Median	39.0	
	25% quartile	32.0	
Years since graduated	Median	13	
	25% quartile	7	
Years working in KSA	Median	6	
	25% quartile	3	
OPD in a day	Median	22	
	25% quartile	15	
		Number	Percentage
Gender	Male	238	67
	Female	117	33
Nationality	Saudi	3	0.8
	Non-Saudi	352	99.2
Type of care providers	GPs	182	51.3
	Internists	115	32.4
	Family	15	4.2
	Physicians	15	4.2
	Endocrinologists	17	4.8
	Surgeons	3	0.8
	Paediatricians	5	1.4
	Gynaecologists	3	0.8
	Other		
Work place	Hospital	100	28.2
	clinic	255	71.8
Category	Consultant	22	6.2
	Senior Specialist	20	5.6
	Specialist	119	33.5
	Resident	23	6.5
	GP	171	48.2
Proportion of diabetic patient in OPD	less than 25%	165	46.5
	25 to 50%	154	43.4
	More than 50%	36	10.1

### Knowledge

Table 2 shows the eight knowledge-related questions and the participants’ responses. The most incorrectly answered question was related to the changes that diabetes mellitus can cause in the eyes, with only 13.2% (*n* = 47) answering it correctly. Conversely, the timing of screening a patient with newly diagnosed type 2 diabetes for DR was known to most of the participants (*n* = 279, 78.6%). The knowledge about DR screening and management was of an excellent level in 1.1% of participants (*n* = 4), good in 53.2% of participants (*n* = 189), and poor in 45.6% of participants (*n* = 162). Good + excellent knowledge was recorded in 193 participants [54.3% (95% CI 49.2–59.5)].

**Table 2:** Knowledge related questions and responses of the participants

No	Questions	Correct answers		Wrong answers		The correct answers
		Number	Percentage	Number	Percentage	
1	A 50-year-old patient is first time diagnosed to suffer from D.M type 2, when should his retinal examination be done?	279	78.6	76	21.4	Soon after diagnosis
2	If your diabetes patient was told by eye doctor that he/she does not have diabetic retinopathy. When should be his/her next diabetic retinopathy (DR) screening?	242	68.2	113	31.8	1 year
3	A10 year-old child is diagnosed to have insulin dependent D.M (Type 1). When should you send him/her for diabetic retinopathy screening?	119	33.5	236	66.5	Within 3–5 years
4	How does a diabetes Patient usually describe lost vision secondary to DR?	275	77.5	80	22.5	Gradual and painless
5	The risk of diabetic retinopathy is much higher and more serious in diabetic patient of long duration in which of the following complication of diabetes?	160	45.1	195	54.9	Diabetic nephropathy
6	Measures that can help in reducing the progression of Diabetic Retinopathy	102	28.7	253	71.3	Healthy lifestyle (regular exercise, good diet & stop smoking), Stringent blood lipid control and Control of systemic blood pressure
7	Diabetes Mellitus can cause the following changes in eye	47	13.2	308	86.8	Macular edema, Cataract formation, Macular ischemia andVitreous haemorrhage
8	Diabetes Mellitus can cause changes in eye which of following changes require to emergency review by ophthalmologist	108	30.4	247	69.6	Sudden loss of vision, pre-retinal or vitreous haemorrhage and Retinal detachment

### The implication of demographic factors on KAP

We correlated the levels of KAP with the demographic determinants in Table 3. Working in a hospital and male gender were found to be marginally related to a higher level of KAP for DR screening and management among the participants.

**Table 3:** Factors related to good practices, positive attitude and good knowledge regarding diabetic retinopathy

		Knowledge			Attitude			Practice		
		Good	Poor	Validation	Positive	Not positive	Validation	Good	Poor	Validation
Gender	Male	125	113	0.3	78	159	0.4	95	141	0.55
	Female	68	49		33	82		50	65	
Place of work	Clinics	47	53	0.08	38	62	0.09	36	109	0.2
	Hospitals	146	109		73	182		64	146	
Type of specialists	GPs +	111	86	0.2	58	138	0.5	83	66	0.2
	Family									
	Physicians	78	80		53	106		103	107	
	Other									

### Barriers to applying DR screening

The participants’ perceived barriers to applying DR screening are depicted in Table 4. A lack of resources (*n* = 267, 75.2%) followed by lack of adequate training (*n* = 245, 69%) were the barriers most recognised by the participants.

**Table 4:** Barriers for DR screening as perceived by non-ophthalmic health care providers

No	Practice related Questions	Yes		No		Not sure	
		Number	Percentage	Number	Percentage	Number	Percentage
1	Busy in other health issues	194	54.6	107	30.1	54	15.2
2	Lack of resources	267	75.2	67	18.9	21	5.9
3	Policy for PHC conflict with mydriasis at PHCs	170	47.9	97	27.3	88	24.8
4	Lack of adequate training	245	69.0	78	22.0	32	9.0
5	Forgot how to examine retina	176	49.6	133	37.5	46	13.0
6	Fear of precipitating glaucoma in patients	158	44.5	120	33.8	77	21.7
7	Non-cooperation of diabetic patients	123	34.6	112	31.5	120	33.8
8	Gender issue for DR screening	154	43.4	100	28.2	101	28.5

Feedback regarding the capacity building of non-ophthalmic eye care professionals in DR management suggested that 80.3% of participants (*n* = 285) believed that they needed hands-on training for DR screening, and 89.6% of participants (*n* = 318) suggested workshops on eye care in diabetes.

## Discussion

The level of knowledge among non-ophthalmic physicians in the private sector concerning people with diabetes was less than desired, as nearly half of the participants had poor knowledge. The attitude for DR screening was found to be positive in one third of participants, which is a problem in that it may lead to the deterioration of the vision of patients with DR. The practice for preventive, by refereeing people with diabetes for early DR detection and following-up with patients after STDR management by an ophthalmologist, was promising in half of the participants (as shown in Table 5). The level of KAP of physicians of the private sector in our study varied according to their work place, which implicates the role of the institutions in the practice of doctors, as some have restrictions while others have good regulations. The participants felt an urgent need for training and creating a protocol for the early detection and management of DR, which is a good indicator for improvement.

**Table 5:** Practice related responses of non-ophthalmic health care professional for DR screening

No	Practice related Questions	Good		Not good		Not sure		The correct answers
		Number	Percentage	Number	Percentage	Number	Percentage	
1	Mydriatic eye drops well maintained at Unit	139	39.2	151	42.5	65	18.3	Yes
2	Ensure working condition of ophthalmoscope	0	0.0	144	40.6	201	56.6	Yes
3	Refer all cases for DR screening	161	45.4	22	6.2	10	2.8	Yes
4	Follows the referred diabetic to know DR status	283	79.7	35	9.9	37	10.4	Yes
5	Follows patients treated for Dr by eye doctor	307	86.5	27	7.6	21	5.9	Yes

A large number of people with DM need to be screened annually for DR in the Kingdom of Saudi Arabia to stop the progression of DR. In the view of ophthalmologists, one of the strategies to reach this goal is to shift the task of early detection to non-ophthalmic physicians [9]. This is possible only if the primary health care physicians are trained and have positive attitude concerning taking up this challenge [11]. Also, physicians of primary health care should counsel the patients regarding their cooperation for undergoing annual DR screening and the timely management of STDR, if the ophthalmologist recommends. Care for diabetes had been shown to improve when counselling is completed by the patients’ attending doctors along with the management [12]. Hence, proper knowledge among physicians about diabetes eye complication and their relationship to visual disabilities is essential.

An excellent level of knowledge was documented in very few physicians in the present study. This was also noted in other studies of government institutions of Saudi Arabia [9], Oman [13], rural China [14] and South Africa [15]. Practitioners’ busy schedules and non-sufficient continued medical education could be the causes for this lack of knowledge regarding DR [16]. One of the areas that can be improved upon is the continuous medical education to general practitioners, which has been demonstrated might improve the management of chronic non-communicable diseases, including diabetes [17]. Additionally, the present study’s results showed that half of participants possessed good knowledge, however, regarding the practice of referrals, only 40% of patients with diabetes were referred for eye screening. The physicians of primary health care who were working in governmental sector of Riyadh had a knowledge score of 57% ±14 and the correct practice of referring patients with Type I diabetic to the ophthalmologist was only 24% [9]. Thus, training and health promotion of health for non-ophthalmic physicians appears to be equally important for both private and governmental sector primary health care physicians.

As in the present study, there is also a weakness in knowledge concerning following up with patients with diabetes for DR screening and the relationship of diabetic duration with the development and progression of DR. However, this study was conducted with final year medical students in Saudi Arabia, which indicates a weakness in their basic clinical knowledge [7]. In another study, conducted in Oman, knowledge of the basic eye structure was satisfactory in only half of physicians, and the practice of fundus examination for DR was poor in 40% of physicians [13]. In practice, 20 physicians had attempted to use an ophthalmoscope, but only 9 could see the details of the retina; so, they also recommend detailed training for their physicians [13]. Moreover, a study was completed in China that stated that among 22 physicians, knowledge regarding eye complications in diabetes was generally good, but physicians did not favour routine pupillary dilation to detect asymptomatic disease; they expressed concerns about workflow as well as the danger and inconvenience to the patients [14]. Further, research from Cape Town, South Africa revealed deficiencies in training, resulting in consequent gaps in knowledge and practice regarding eye complications in diabetes [15].

Therefore, special focus is needed to improve knowledge about tele-screening for DR. A positive attitude towards eye care and laying down a protocol will ensure a uniform practice for DR screening and STDR management. Such recommendations have been periodically issued in Middle Eastern countries, as diabetes in Arabic-speaking countries is poorly controlled and the risk of diabetes complications in the eyes is high [18, 19].

## Conclusions

Improving KAP level is urgently needed. Addressing underlying causes of low KAP could enhance the eye care of patients with diabetes. Furthermore, training for primary health care providers regarding the early detection of DR and timely management of STDR is necessary.

## Data Availability

The datasets used for the current study are not publicly accessible due to ethical reasons but are available from the corresponding author on reasonable request.

## Abbreviations

DM: Diabetes Mellitus
DR: Diabetic Retinopathy
IRB: Institution Review Board
KAP: Knowledge, Attitude, Practice
PHC: Primary healthcare
STDR: sight threatening diabetic retinopathy
